# Retraction: Overexpression of circ_0034642 contributes to hypoxia-induced glycolysis, cell proliferation, migration and invasion in gliomas by facilitating TAGLN2 expression *via* sponging miR-625-5p

**DOI:** 10.1039/d1ra90061g

**Published:** 2021-02-01

**Authors:** Laura Fisher

**Affiliations:** Royal Society of Chemistry Thomas Graham House, Science Park, Milton Road Cambridge CB4 0WF UK advances-rsc@rsc.org

## Abstract

Retraction of ‘Overexpression of circ_0034642 contributes to hypoxia-induced glycolysis, cell proliferation, migration and invasion in gliomas by facilitating TAGLN2 expression *via* sponging miR-625-5p’ by Bo Kong *et al.*, *RSC Adv.*, 2020, **10**, 897–908, DOI: 10.1039/C9RA08600E.

The Royal Society of Chemistry hereby wholly retracts this *RSC Advances* article due to concerns with the reliability of the data. The images in the article, and the raw data provided by the authors, were screened by an image integrity specialist.

The western blots in this paper all have the same look, the bands have a very generic shape on very similar-looking backgrounds, and do not look genuine. The raw data provided had no background apart from light reflexes that may have been added artificially. Further analysis of the raw data provided for Fig. 5F and G, revealed that the GAPDH control panels in both figures were identical. Furthermore, the background to the TAGLN2 panels were identical, but the bands are not. The raw data, therefore, does not appear to be genuine and cannot be used to validate the published data.

Given the significance of the concerns about the validity of both the data in the article and the raw data provided by the authors, the findings presented in this article are not reliable.

Fujun Wang opposes the retraction. The other authors have been informed but have not responded to any correspondence regarding the retraction.

Signed: Laura Fisher, Executive Editor, *RSC Advances*.

Date: 19^th^ January 2021.

## Supplementary Material

